# Intraoperative Computed Tomography versus Perdriolle and Scoliometer Evaluation of Spine Rotation in Adolescent Idiopathic Scoliosis

**DOI:** 10.1155/2015/460340

**Published:** 2015-03-10

**Authors:** Rafal Pankowski, Szymon Wałejko, Marek Rocławski, Marcin Ceynowa, Tomasz Mazurek

**Affiliations:** Department of Orthopaedics, Medical University of Gdansk, Nowe Ogrody 1-6, 80-803 Gdansk, Poland

## Abstract

Numerous indirect methods for apical vertebral rotation (AVR) measurement have been reported and none of them seems to be as accurate as computed tomography evaluation. The aim of this study was to compare spinal rotation changes during innovative technique of intraoperative computed tomography (ICT) evaluation with indirect methods such as Perdriolle and clinical evaluation with scoliometer. We examined 42 adolescent idiopathic scoliosis (AIS) patients treated with posterior scoliosis surgery (PSS). The mean age at the time of surgery was 16 years. ICT evaluation was performed before and after scoliosis correction in prone position. Clinical rib hump measure with scoliometer and radiographic Perdriolle were performed before and after surgery. There was 71,5% of average rib hump correction with scoliometer but only 31% of correction with ICT (*P*=0,026) and there was no significant correlation between them (*R*=0,297, *p*=0,26). Mean postcorrectional Perdriolle AVR had a decrease of 16,5°. The average ICT AVR had a decrease of only 1,2° (*P*=0,003). There was no significant statistic correlation between ICT and Perdriolle AVR evaluation (*R*=0,297, *p*=0,2). There is a significant discrepancy in AVR and rib hump assessment between scoliometer and Perdriolle methods and ICT evaluation, which seems to be the most accurate tool for spinal derotation measurement.

## 1. Introduction

The principal goal of adolescent idiopathic scoliosis (AIS) surgery is to stop the deformity progression to correct it with instrumentation and spondylodesis. The present evolution of posterior scoliosis surgery (PSS) has focused mainly on apical derotation improvement. The first method of intraoperative correction of rotational deformity with the turning of the rod opposite to the direction of rotation was introduced by Cotrel and Dubousset and was called “derotation on the rod.” After some time, the original Cotrel-Dubousset (CD) method based on hook systems only was substituted with “segmental pedicle screw only” constructs. The latest spinal implant technology allows intraoperative direct vertebral derotation (DVD). Surgical techniques used in our hospital are the reflection of global trends in scoliosis surgery. The original CD method with hooks was first introduced in our clinic in 1998, modified with all pedicle screw constructs in 2006 and DVD systems since 2008. Although there are numerous methods of spinal axial rotation measurements, the majority of them are indirect techniques, which use landmark identification such as Cobb method based on spinal processes positioning [1]. In general indirect radiographic methods are limited to the measurement of only two spinal planes: sagittal and coronal. On the other hand, direct methods are based on three-dimensional technology (axial, coronal, and sagittal planes, computed tomography (CT)). Subsequent studies on spinal rotation were performed by Nash and Moe. Scoliometer clinical evaluation is the simplest indirect method of spinal rotation assessment during the Adams Forward Bending Test (AFBT). Thoracic rib hump (TRH) and lumbar muscular wall (LMW) measurements performed with scoliometer (Bunnell) can indirectly indicate the apical vertebral rotation (AVR). Computed tomography (CT) evaluation described by Aaro and Dahlborn in 1981 is the most accurate tool for spinal rotation measurement (SRM) due to the currently best bone visualization. However, the pre- and postoperative CT scans do not provide precise data due to patient positioning and the rib hump pressure against the CT table during two subsequent measurements as well as irregular reference points in spinal rotation assessment. Considering these facts CT evaluation in prone position of the patient seems to be more accurate than in supine position [2]. One can achieve perfect conditions for SRM intraoperatively when both the reference points and the patient's position are constant before and after corrective maneuvers.

## 2. Aim

The aim of this study was to verify the true spinal derotation during intraoperative computed tomography (ICT) and to compare direct SRM with indirect methods (Perdriolle and scoliometer evaluation) in AIS patients treated with PSS.

## 3. Material and Methods

**Figure 1:**
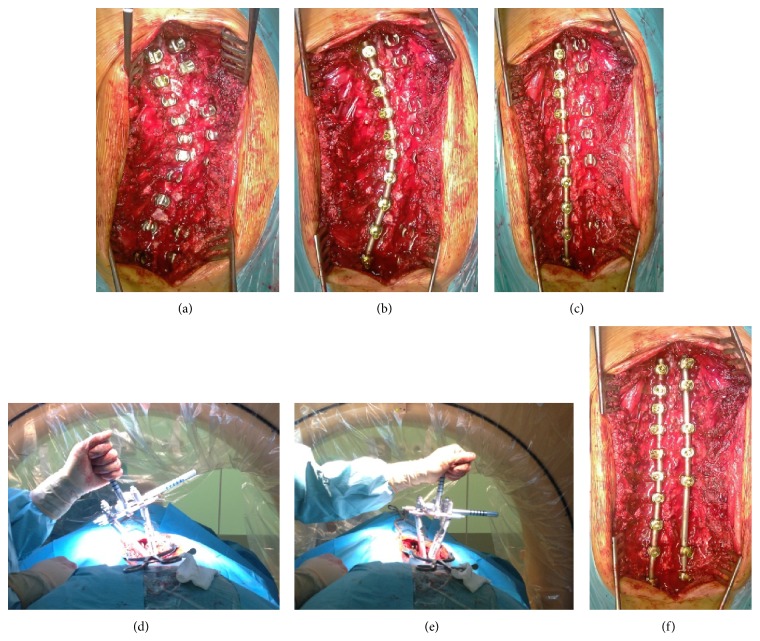
Steps of the surgical correction, (a) screw placement, (b) rod contoured to the curve, (c) after “derotation on the rod,” (d) before DVD, (e) after DVD, and (f) second rod on the convex side.

A consecutive group of 42 AIS patients (36 females and 6 males) was examined. The average age at the time of surgery was 16 years (12,4–18 years). Female to male ratio was 6 to 1. All curves were described according to the Lenke classification. Among 42 evaluated patients, 24 had single curve thoracic scoliosis (Lenke type 1), 2 patients double thoracic (Lenke type 2), 4 with double major thoracolumbar curve (Lenke type 3), and 12 patients had thoracolumbar curve (Lenke type 5). There were no Lenke types: 4 and 6 of spinal deformity in the study group. All patients were treated with PSS with all pedicle screw constructs. Two separate corrective maneuvers were proceeded in every case such as derotation on the rod and DVD (vertebral column manipulation (VCM), Medtronic) (Figure 1).

**Figure 2:**
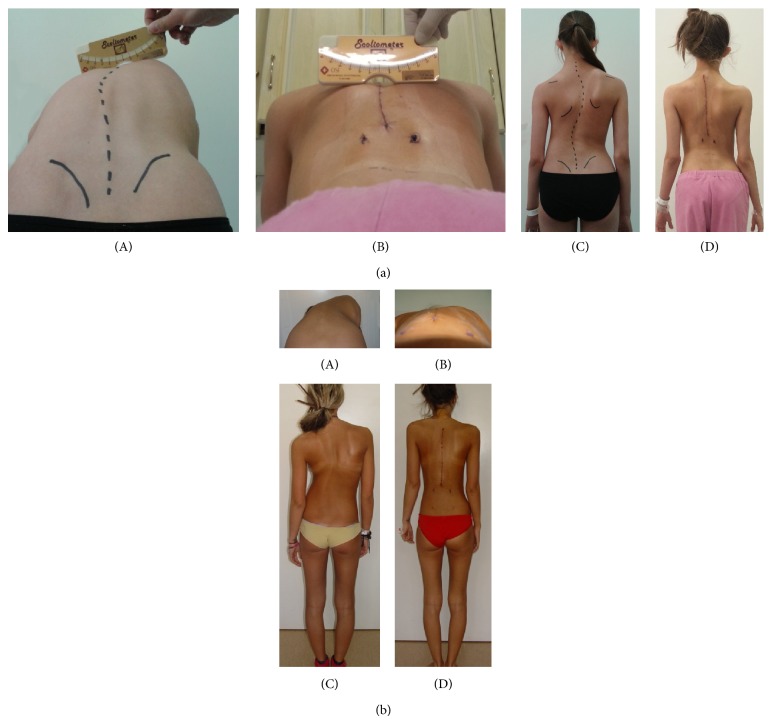
(a) “Patient 2:” (A) pre- and (B) postoperative AFBT with Bunnell scoliometer. Standing position (C) before and (D) after correction. (b) “Patient 27:” (A) pre- and (B) postoperative AFBT. Standing position (C) before and (D) after correction.

**Figure 3:**
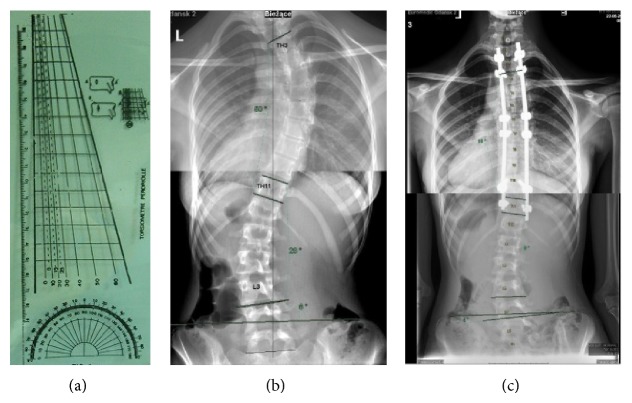
(a) Perdriolle torsionmeter, (b) pre- and (c) postcorrectional Cobb angle measurement on plain radiographs.

Indirect spinal rotation changes were evaluated pre- and postoperatively during clinical AFBT and radiographic Perdriolle method. AFBT was performed with Bunnell scoliometer and measurements were taken at the apex of the rib hump or lumbar muscular wall (Figure 2). Pre- and postoperative apical vertebral rotation (AVR) measurements were performed with Perdriolle torsionmeter on PA radiographs according to the original Perdriolle method. Pre- and postoperative Cobb angle measurements of curve magnitude on PA radiographs also were performed [1] (Figure 3).

**Figure 4:**
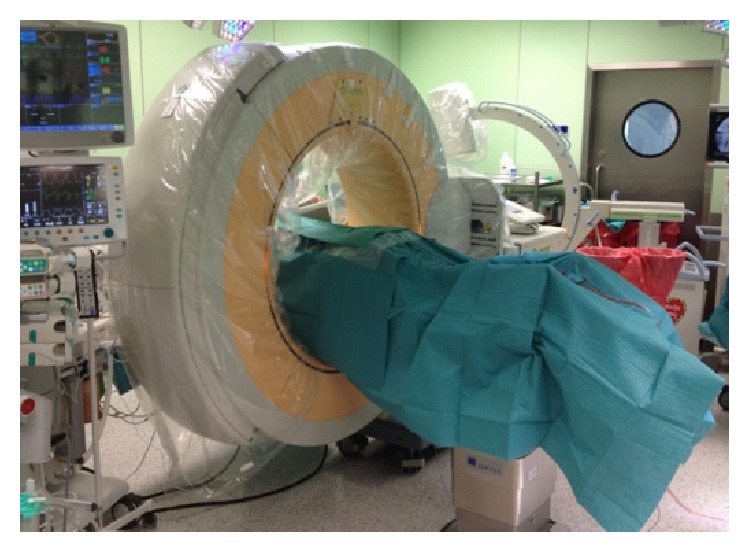
Intraoperative computed tomography evaluation with O-Arm (Medtronic).

A direct AVR measurement was performed with ICT evaluation (O-Arm, Medtronic) before and after scoliosis correction. All patients remained in prone position during these examinations (Figure 4).

**Figure 5:**
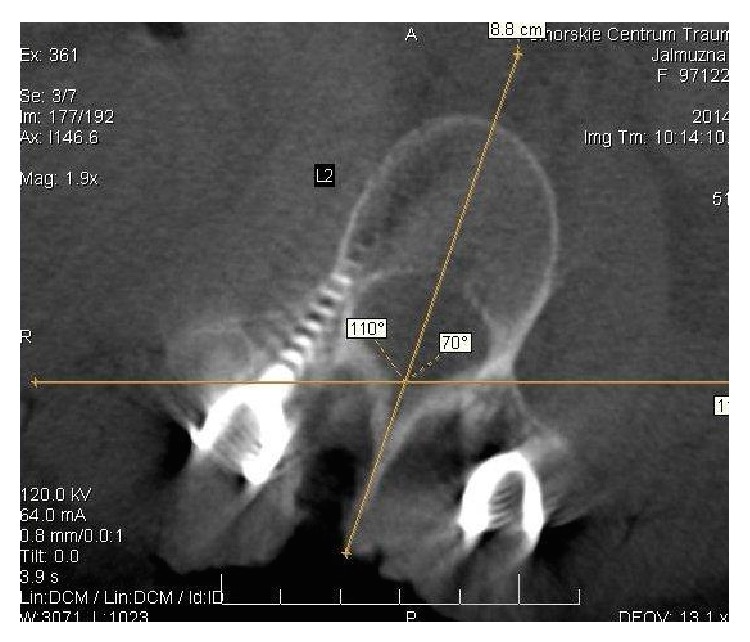
ICT evaluation AVR measure with Aaro and Dahlborn (A&D) method.

The first and the last AVR measurements were taken into consideration. During the first ICT, AVR measurement with Aaro and Dahlborn (A&D) method was performed (Figure 5).

**Figure 6:**
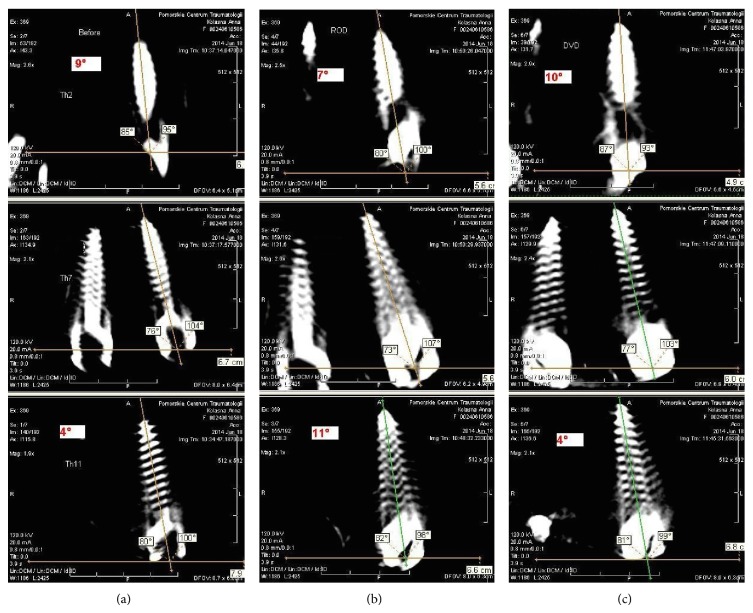
A new method of derotation assessment on the CT scans, two-step procedure. (a) Before correction, (b) after rod derotation, and (c) after DVD. Upper window: upper instrumented screw (UIS), middle window: apical screw (AS), and lower window: lower instrumented screw (LIS). The longitudinal axis of the particular screw was marked which crossed the middle of the head, the body, and tip of the screw. This line created an angle with the horizontal reference line. The difference of these angles between AS and UIS and AS and LIS before and after surgery allowed the real apical vertebral derotation (AVD) assessment. AS (°)-UIS (°): bigger windows in upper section, AS (°)-LIS (°): bigger windows in the lower section.

Due to the difficulties in determination of reference points for AVR measurement in A&D our own method of SRM was performed on the pre- and postcorrectional ICT scans. In our opinion pedicle screws can be constant reference points for derotation assessment. It is also easier to visualize the screw contour than the vertebral body contour in A&D method. The longitudinal axis of the screw in the apical vertebra (AV) is generally different than AVR value, whereas the difference of the angles between the longitudinal axes of screws in AV and upper instrumented vertebra (UIV) and lower instrumented vertebra (LIV) enable the evaluation of changes in AVR. Pedicle screws in the apical vertebra, UIV, and LIV were marked as a constant reference points and were tagged as apical screw (AS), upper instrumented screw (UIS), and lower instrumented screw (LIS), respectively. Only the scans with accurate screw outline were chosen for the analysis. The longitudinal axis of the particular screw, which passed over the middle of the head, trunk, and top of the screw, was marked. This line created an angle with the horizontal reference line, which was set down by the X-ray evaluation software. The difference of angles between AS and UIS and AS and LIS before and after surgery allowed the real apical vertebral derotation (AVD) assessment (Figure 6). If the distance between tips of two screws in AV after correction was different than before correction, we marked it as an implant loosening and excluded from further assessment because this could influence the outcome of evaluation due to the change in longitudinal axes of screws. In case of no change in the distance between tips of two screws before and after correction we recognized the screw rotation as equivalent with vertebral rotation. Eventually we used the following mathematic formulas to calculate parameters below:

(1) AVR  precorrectional( °)=A&D( °),AVD=α°−β°,α°=AS  postcorrectional( °)−UIS  postcorrectional( °)  + AS  postcorrectional °−LIS  postcorrectional ° ·2−1,β°=AS  precorrectional( °)−UIS  precorrectional( °)  + AS  precorrectional °−LIS  precorrectional ° ·2−1.

Decrease of mean angle between screws (**α**° − **β**°)  1° or more indicated the true spinal derotation and the result had negative value (AVD = −*n*°). Positive AVD values indicated the increase of mean angle between screws and increase of postcorrectional AVR. The final postcorrectional AVR value was established with the formula below:

(2) $$\begin{array}{c} \text{AVR}\;\text{postcorrectional}\left({\;}^{{}^{\circ}}\right)=\text{A}\&\text{D}\left({\;}^{{}^{\circ}}\right)+\text{AVD}\left({\;}^{{}^{\circ}}\right). \end{array}$$

**Figure 7:**
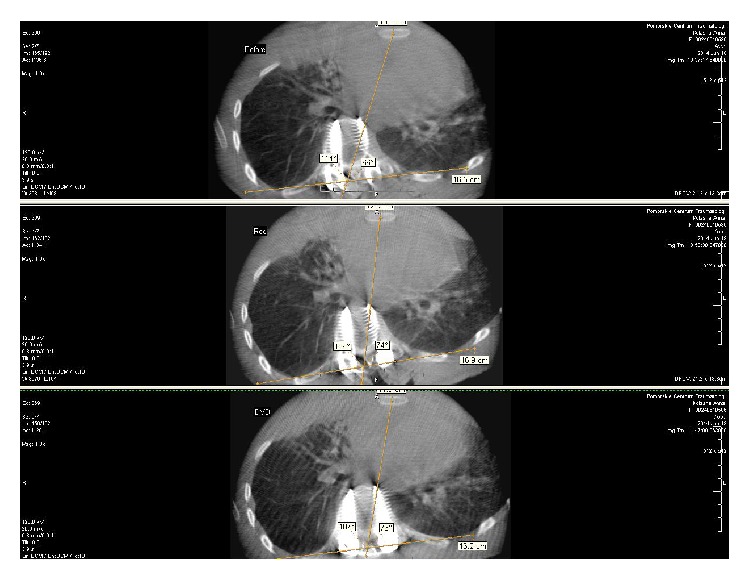
Rib hump angle ICT measurement (pleura-pleura (P-P) line and spine-sternum (S-S) line ratio). P-P line was marked between the two highest points of pleura at the apex of the curve. S-S line was marked between the middle of the basis of spinal process of apex vertebra with the midline of the sternum.

Other evaluated ICT parameters included pre- and postcorrectional rib hump angle (pleura-pleura (P-P) line and spine-sternum (S-S) line ratio). P-P line was marked between the two highest points of pleura at the apex of the curve. S-S line was marked between the middle of the basis of spinal process of apex vertebra with the midline of the sternum (Figure 7). All patients had intraoperative spinal neuromonitoring (ISN) performed. None had the motor evoked potentials (MEP) and the somatosensory evoked potentials (SSEP) disturbed. For statistical analysis SPSS 17v software was used. The Student *t*-test was used for the examination of statistical differences. *R*-Spearman test was used for correlation assessment.

## 4. Results

**Figure 8:**
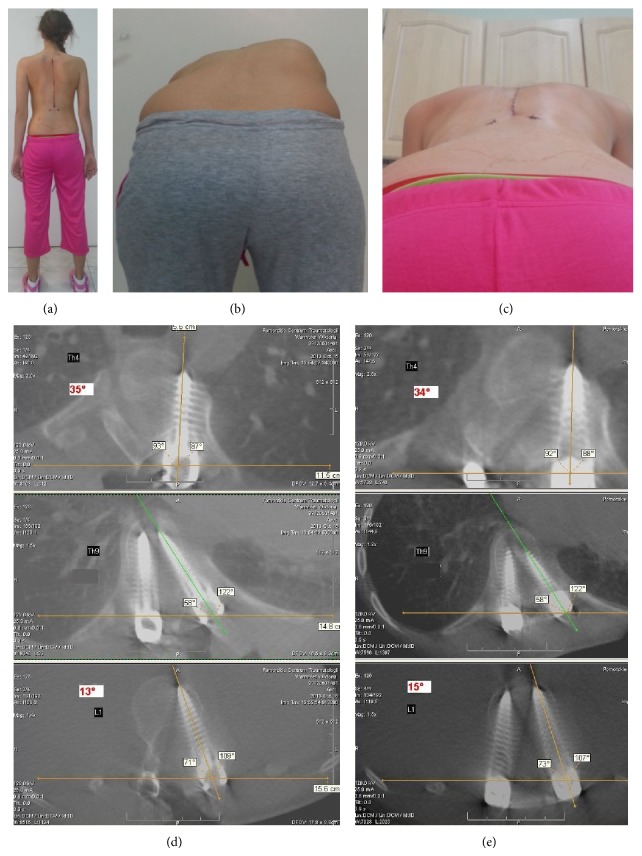
“Patient 13:” (a), (b), and (c) good clinical outcome. (d) Precorrectional CT scan of the same patient: upper window (UIS), middle (AS), lower window (LIS), AS  ( °) − UIS  ( °) = 35° (bigger window in upper section), AS  ( °) − LIS  ( °) = 13° (bigger window in the lower section). (e) Postcorrectional CT scan worsening of AVR after correction, AS  ( °) − UIS  ( °) = 34°, AS  ( °) − LIS  ( °) = 15°, AVR  precorrectional  ( °) = A&D  ( °) = 26°, AVD = (34° ± 15°)/2 − (35° ± 13°)/2 = 0,5°, AVR postcorrectional (°) = 26° + 0,5° = 26,5°. Poor ICT outcome in contrast with Perdriolle AVR.

We excluded from the study group from further assessment two patients into whom the distance between tips of two screws in AV after correction was different than before correction, what we marked as an implant loosening. Eventually 40 patients left for further evaluation. Mean precorrectional thoracic and lumbar curve Cobb angle were 49° ± 15° and 38° ± 11,3°, respectively. Postcorrectional thoracic and lumbar Cobb angle were 15° ± 5,7° and 7° ± 7°, respectively. The mean correction rate of the thoracic curve was 69% and of the lumbar curve was 81% (Table 1). A mean pre- and postcorrectional rib hump measurement with scoliometer was 14° ± 4,5° and 4° ± 2,9° (71,5% correction), respectively. Mean pre- and postcorrectional rib hump ICT measures were 23° ± 6,5° and 16° ± 6,4°, respectively (31% correction). There was a statistically significant difference in correction rates between clinical and ICT evaluation (*P* = 0,026), and there was no significant correlation between them (*R* = 0,297, *p* = 0,26). Mean pre- and postcorrectional Perdriolle AVR were 22° ± 7,6° and 9° ± 6,9° in thoracic (59% correction) and 27° ± 7,3° and 10° ± 5,9° in lumbar spine (63% correction), respectively. Mean postcorrectional Perdriolle AVR decreased of 16,5°. Mean precorrectional Aaro and Dahlborn (A&D) AVR were 13,5° ± 4,2° and 16,3° ± 11° for thoracic and lumbar spine, accordingly. Mean pre- and postcorrectional angles between AS and UIS were 12,3° ± 8,4° and 10,1° ± 7,8° and between AS and LIS 8,7° ± 7,1° and 8,5° ± 7,4°, accordingly. The mean ICT-apical vertebral derotation (AVD) was only 1,2° in contrast to the AVD assessed with Perdriolle method (*P* = 0,003). There was a true AVD (≥1°) in 18 patients in contrast to 12 with no change in AVR and 6 with increased AVR after corrective maneuvers. In 4 patients with double curve scoliosis (both curves instrumented) there was an improvement in one but deterioration in the second curve. There was no significant statistical correlation between CT and Perdriolle AVR evaluation (*R* = 0,297, *p* = 0,2) (Figure 8).

## 5. Discussion 

Although the progression restraint is the primary goal of the operative treatment of AIS, patient's expectancies are higher and more directed towards better cosmesis. The maximal 3D correction with particular emphasis on AVR control can have a positive effect on the reduction of rib hump deformity. In consequence, this can improve the patients' quality of life (QoL) [3]. With both the scoliometer and the ICT there was a significant rib hump reduction. However, the degree of improvement was significantly better in clinical (71,5%), than in ICT evaluation (31%). There were no significant statistic correlations between ICT and Perdriolle methods of AVR evaluation (*R* = 0,297, *p* = 0,2). It leads us to the conclusion that one should not use these two methods alternatively, because one scale cannot predict the result of the other. The measurement differences between both methods can be influenced by the fact that during clinical assessment with the scoliometer the outer contours of the thorax are affected by the thickness of the soft tissues of the thoracic wall. In ICT evaluation, the inner contour of the thorax with reference points such as the spine, the sternum, and the highest points of pleura on both sides are measured. Due to the patient's expectations of rib hump decrease we propose to use a scoliometer in clinical examination for outcome measure and ICT scans for objective review. True spinal derotation in AIS treatment is one of the most difficult objectives to achieve. The diversity of DVD systems can contribute to this fact. Current “in vivo” physiological studies of spinal rotation emphasize its limited range and association with the lateral bending of the scoliotic curve. As a consequence, spinal derotation can be both limited and difficult to measure. Moreover, a rib hump can be a result of not only the rotation of particular vertebrae but also the result of the rotation of the whole spine “en bloc” and the torsion of particular vertebrae. For these reasons the improvement of the shape of the thorax is influenced by a multitude of maneuvers: the translation, distraction, and rotation between both the thoracic and lumbar curves. This phenomenon can imitate the true spinal derotation when indirect methods of its assessment are used. The results obtained by Cobb, Nash and Moe, Perdriolle, Stokes or Mehta are all based on the analysis of classic radiographs and clinical assessment of the patient [1, 4–7]. Meanwhile, the precise measurement of spinal rotation can be a crucial point in the outcome prognosis and the choice of the method of treatment [8–10]. Additionally, it can be used as an indicator of the curve progression risk during both pre- and postoperative assessments of the AIS patients [11]. Clinical rib hump evaluation as an indirect spinal rotation measurement is routinely used in schools among children and adolescents [12]. An accurate SRM is crucial for proper placement of pedicle screws and to the decrease of risk of spinal cord injury [13]. It can also help in better understanding of derotative effect of Cotrel-Dubousset instruments in comparison with the Harrington method [14–18]. The methodological breakthrough in the spinal rotation assessment was the introduction of the Aaro-Dahlborn CT method [19]. It has been recognized to be a method of high quality and is acknowledged by many orthopaedic surgeons. In their study, Ho et al. came to a conclusion that neutral positions of vertebras measured with Nash and Moe method were in fact in 11° of rotation [20]. Kaczmarczyk showed even a greater disproportion between CT and indirect methods, with differences of up to 20° [21]. Despite these facts CT evaluation of spinal rotation can be imperfect. The measurement error of the single CT evaluation is around 2° and rises to 4° when two different scans are compared [10]. The possible cause of this can be a lack of direct anatomical reference points specification and the presence of implant artifacts. Additionally, the measurement error can be amplified by the patient's different pre- and postoperative positions (prone and supine) and the effect of chest compression against the CT table. Considering these facts we developed our own method of spinal rotation and derotation assessment. What is worth to emphasize is that the patient stays in the same prone position throughout the whole procedure both before and after spinal derotation. This eliminates the effect of the table compression against the rib hump and the other position changes during the two following evaluations. The importance of patient's positioning was stated by Abul-Kasim et al. [2]. Another original solution in our study protocol was the introduction of direct reference points which included the longitudinal axes of the pedicle screws placed in apical vertebrae and upper and lower instrumented vertebras. This method can decrease the error ratio during the single assessment up to 2° which is dictated by the computer software. Our derotation assessment method showed weak points of the “derotation on the rod” maneuver that was considered the basis of the original CD method. This study showed that the “derotation on the rod” maneuver failed and even it increased the AVR and the rib hump size in a few patients. Krismer et al. in their CT study did not observe significant spine derotation as well [22]. Gray et al. came into similar conclusions when examined CD patients [23]. Kaczmarczyk showed that the true derotation (CT) with the CD method is minimal and even close to the measurement error [21]. On the other hand, Labelle et al. claimed that the effectiveness of CD system is based on the effect of relocation of the curve “en bloc” rather than the true derotation between particular vertebras which is concurrent with our observations [24]. In our study the DVD method was not always effective not in every case. The most surprising fact was the discrepancy between the lack of true spinal derotation and a good silhouette of patients. The rib hump decreased significantly in every case after the surgery and the silhouette came to be close to the physiological state. Despite the obvious good clinical correction ICT evaluation showed very limited spinal derotation. Considering the ICT evaluation is very accurate method for AVR assessment, the Perdriolle method and other indirect methods seem to be very limited and approximate. Significant discrepancies between mean derotation values in Perdriolle method do not allow its use routinely for the true spinal rotation changes after corrective maneuvers. ICT derotation measurement seems to be an optimal solution according to our experience. It will be even more reliable if interobserver and intraobserver reliability of the new measuring method on ICT can be tested in our center in the near future. An increased radiation dose during this method can be a slight limitation of the method, but on the other hand an assessment of proper and safe pedicle screws placement is made possible with ICT, as well as recognizing possible vertebral body injury or implant loosening. The mean absorbed dose of radiation in our study was comparable with standard fluoroscopy. Other limitations of this study must also be considered. It is not directly comparable between the Perdriolle method and scoliometer measurement with A&D or the new ICT method proposed, because they are based on different measuring theories and techniques. Measuring time (intraoperation versus pre- and postoperation), measuring posture (prone versus standing or forward bending), and patient's condition (anesthesia versus consciousness) might be confounding factors which contribute to result discrepancy. This direct comparison can be difficult to conduct in real, but on the other hand there were many prior studies based on indirect SRM and it is worth to refer the ICT outcome to them. Above all the aim of this study was to objectively identify the true spinal derotation during ICT evaluation and additionally compare it with AVD evaluated with indirect methods (Perdriolle and scoliometer evaluation) in the study group. Due to the significant discrepancies between these methods, future studies on this issue must be provided.

## 6. Conclusions

Consider the following.

1. The true spinal derotation during AIS surgery even with DVD methods is slight and possible to assess precisely only when CT evaluation is used.
2. There is a significant discrepancy in spinal derotation assessment between indirect methods (scoliometer, Perdriolle) and objective ICT evaluation.
3. Indirect spinal derotation measurements (clinical and radiographic) are not suitable for specification of mechanisms responsible for a rib hump decrease.
4. The apparent derotation (chest contour improvement and rib hump decrease) is perhaps possible to obtain through translation of the apical vertebrae to the midline, but more literatures and supports are necessary to discuss such hypothesis of “coupling effect” of coronal and sagittal plane.
5. Intraoperative CT seems to be the accurate tool for spinal derotation measurement.

## Figures and Tables

**Table 1:** Comparison of pre- and postcorrectional clinical and radiographic parameters evaluated with direct and indirect methods of measurement.

Parameter	Evaluation	
	Before correction	After correction
Mean Cobb angle (°) thoracic curve	49,0° ± 15°	15° ± 5,7°
Mean Cobb angle (°) lumbar curve	38,0° ± 11,3°	7° ± 7°
Mean curve correction ratio (%) thoracic curve		69%
Mean curve correction ratio (%) lumbar curve		81%
Mean rib hump high (°) scoliometer	14° ± 4,5°	4° ± 2,9°
Mean rib hump correction ratio (%) scoliometer		71,5%
Mean rib hump high (°) ICT	23° ± 6,5°	16° ± 6,4°
Mean rib hump correction ratio (%) ICT		31%
Mean thoracic AVR (°) PERDRIOLLE	22° ± 7,6°	9° ± 6,9°
Mean lumbar AVR (°) PERDRIOLLE	27° ± 7,3°	10° ± 5,9°
Mean thoracic AVR (°) A&D	13,5° ± 4,2°	
Mean lumbar AVR (°) A&D	16,3° ± 11°	
Mean (AS – UIS) angle (°)	12,3° ± 8,4°	10,1° ± 7,8°
Mean (AS – LIS) angle (°)	8,7° ± 7,1°	8,5° ± 7,4°
Mean AVD (°) PERDRIOLLE		15°
Mean AVD (°) DVD ICT		1,2°
